# Muscle Strength and Power: Primary Outcome Measures to Assess Cold Water Immersion Efficacy After Exercise With a Strong Strength or Power Component

**DOI:** 10.3389/fspor.2021.655975

**Published:** 2021-06-14

**Authors:** Angus Lindsay, Jonathan M. Peake

**Affiliations:** ^1^Institute for Physical Activity and Nutrition, School of Exercise and Nutrition Sciences, Deakin University, Geelong, VIC, Australia; ^2^School of Biomedical Sciences, Queensland University of Technology, Brisbane, QLD, Australia; ^3^Sport Performance Knowledge and Innovation Excellence, Queensland Academy of Sport, Brisbane, QLD, Australia

**Keywords:** cryotherapy, recovery, performance, muscle damage, exercise, excitation contraction coupling

## Introduction

Recovery from exercise-induced muscle damage, fatigue or stress is critical for restoration of exercise performance. In most exercise activities or sports, performance is regulated through the physiological capacity of muscle. Therefore, athletes, coaches, and scientists have continued to explore post-exercise recovery modalities that focus on expediting muscle functional recovery. Several post-exercise recovery strategies have been developed, tested, and used in amateur and professional athletes to expedite muscle functional recovery. A cost-effective and well-researched practice to achieve this goal is cold water immersion (CWI). CWI requires submersion of a limb or the whole body in cold water of a specified temperature (usually <15°C) for a specified duration immediately post-exercise, or over several succeeding days. However, the equivocal findings on the efficacy of CWI and the extensive number of outcome variables has made it challenging to interpret and correctly implement this intervention. In this short opinion piece, we briefly review CWI research and the challenges that practitioners and athletes face when deciding whether to use CWI as a post-exercise recovery intervention. We then discuss why muscle strength and/or power should be considered the primary outcome variable in CWI research with a strong strength and power component, and why excitation–contraction coupling and/or rate of force development assessment is necessary to evaluate strength/power-specific changes. Finally, we present systematic evidence that there is a dearth of strength measurements in CWI research, which could be limiting our understanding of this post-exercise recovery strategy.

## A Brief History of CWI Research

Some of the first evidence that a single CWI application might be beneficial for recovery from muscle damage or injury was provided by Hayden (Hayden, 1964) and Hocutt et al. (1982). Both studies showed that CWI expedited return-to-duty in soldiers after injury, or return to full activity after ankle sprain, respectively. In contrast, Matsen et al. and Marek et al. showed that application of cold water to an injury significantly increased oedema (Matsen et al., 1975; Marek et al., 1979). Subsequent research about the effects of CWI has also produced some contrasting findings. For example, CWI immediately following blunt trauma to skeletal muscle of rats significantly reduced oedema formation (Dolan et al., 1997). By contrast, CWI following eccentric contractions did not affect muscle soreness or strength in humans (Eston and Peters, 1999). More recently, Naderi et al. showed that CWI did not attenuate a loss in muscle strength following a single bout of strength training (Naderi et al., 2021), whereas Kodejška et al. (2018) demonstrated that CWI increased the force time integral in handgrip performance compared with passive recovery in rock climbers. Differences in CWI study outcomes could be associated with different modes of exercise, methods or timing of applying CWI and approaches to assessing muscle damage.

Contradictory findings related to repeated CWI applications have also been reported and confirmed in a recent systematic review and meta-analysis (Malta et al., 2021). Fu et al. (1997) showed that when CWI was regularly applied to rats after exercise training, it caused advanced ultrastructural damage to myofibrils. Several human studies have also shown negative adaptive effects of repeated CWI applications after resistance training (Fröhlich et al., 2014; Roberts et al., 2015; Yamane et al., 2015; Fyfe et al., 2019; Poppendieck et al., 2020). However, Lindsay et al. (2016) showed that repeated CWI applied to mixed martial artists during a training camp attenuated the inflammatory response, but did not affect measures of performance. Repeated CWI applied after high intensity interval training (HIIT) or a combination of HIIT with low-moderate intensity aerobic exercise also does not influence indices of performance or muscle cellular signaling (Halson et al., 2014; Aguiar et al., 2016; Christiansen et al., 2018). Although these studies represent only a small proportion of published CWI research, they do demonstrate the complexity in understanding the value of this recovery intervention.

## Interpreting CWI Research

Regardless of the inconsistencies in CWI study outcomes, anecdotal evidence suggests that professional athletes from various sports use CWI as a post-exercise recovery strategy. The reasons behind this persistent practice are uncertain, but may reflect a disconnect between the scientific findings of the studies, and how coaches and athletes interpret these findings. The practitioners' guide to determining if, and when, to implement CWI is confounded by the vast performance, biochemical, and qualitative analyses that have been used to evaluate its efficacy. Other than the variability in CWI protocols (which can range from 4 to 15°C, 5 to 30 min durations, 1 to 10 applications, immediate to delayed submersion) and level of exercise intensity, participant sex and training status, the outcome variables of interest provide an added level of complexity in CWI study comparisons. First, a practitioner or self-coached athlete without in-depth scientific knowledge of biological processes, may not be able to interpret correctly the results of CWI studies that focus on indices of inflammation, gene expression or rates of protein synthesis. Second, drawing comparisons between performance and biochemical or molecular variables could be challenging for a non-scientist. Physiological performance analysis offers a direct and interpretable option for practitioners that is training-specific. We therefore propose that consistently measuring maximal muscular strength and/or power [product of load lifted and angular displacement (distance load moved) divided by time spent moving the load (Sapega and Drillings, 1983; Winter et al., 2016; Horta-Gim et al., 2021)] will provide the exercise community with a more appropriate understanding of whether CWI enhances recovery from exercise-induced damage or fatigue, and improves the performance and work capacity of athletes. From our perspective as exercise physiologists, maintenance of muscular strength and power, irrespective of any changes in muscle ultrastructural integrity, will likely benefit overall physical performance.

## Muscle Strength/Power and Mechanisms of Strength/Power Loss

Muscle strength and power outcomes is a multi-faceted coordination of electrical and chemical events, together with interactions between structural components of muscle tissue. Strength and power are measured using the 1-repetiton maximum (actual or estimated), or with force transducers or plates associated with lab-based dynamometers that measure absolute torque production and rate of force development. Loss of muscle strength associated with eccentric contractions (which lengthen the muscle during simultaneous force production) can be primarily attributed to excitation–contraction uncoupling, and to a lesser extent, loss of contractile protein and structural damage (Warren et al., 2001, 2002). Therefore, post-exercise recovery interventions should target the processes of excitation–contraction coupling to accelerate recovery from eccentric contraction-biased exercise. The triad of skeletal muscle is the site of excitation–contraction uncoupling following eccentric contraction-induced strength loss. More specifically, it is the voltage-sensitive dihydropyridine receptors (DHPR) located in the T-tubules and the ryanodine receptor (RyR) calcium release channel of the sarcoplasmic reticulum (Ingalls et al., 1998; Warren et al., 2001, 2002; Corona et al., 2010; Baumann et al., 2014). The sensitivity of both the DHPR and RyR are not affected by eccentric contraction-induced strength loss (Ingalls et al., 2004a). However, the expression of proteins that associate with the DHPR and RyR to modulate cross-talk and calcium release is significantly decreased (Corona et al., 2010; Baumann et al., 2014). Thus, assessing the effectiveness of CWI for restoring muscle strength could include molecular measurement of the DHPR, RyR, junctophilin, FKPB12, calmodulin, or calsequestrin (proteins associated with the triad of muscle fibers and known to interact with channels and receptors regulating skeletal muscle calcium kinetics). Because cold acclimation can influence calcium handling/kinetics of skeletal muscle and improve indices of muscle performance (Bruton et al., 2010), additional calcium measurements following CWI could supplement analyses of excitation–contraction coupling. However, we do acknowledge that such measurements of proteins following CWI would require time course evaluation, multiple muscle biopsies that would complicate human studies with respect to recruitment and full participation, and confound interpretations of findings by non-scientists.

Researchers can indirectly assess excitation–contraction uncoupling *in vivo* by comparing the low-frequency to high-frequency torque loss prior to and following CWI. The greater reduction in low-frequency torque compared with high-frequency torque indicates excitation–contraction uncoupling (Edwards et al., 1977; Jones et al., 1982; Ingalls et al., 2004b). Cheng et al. (2017) showed that in isolated single muscle fibers of mice, cold application following fatiguing contractions dampened submaximal force without altering maximal force during recovery. In fact, the ratio of submaximal to maximal force was lowest with the coldest temperature, suggesting greater excitation–contraction uncoupling with colder applications. Additionally, we acknowledge that force-generating capacity during rapid, dynamic movements is also relevant to athletic performance and may represent a more sensitive measure to detect changes in neuromuscular function. Rate of force development can generally be determined by measuring the change in peak force divided by a change in time (maximal rate of contraction to accommodate for inter-individual variability in peak force development time) using lab-based force transducers and associated software. Central nervous system (CNS) fatigue likely also influences recovery of muscle strength and/or power following a single or repeated applications of strenuous exercise (Peiffer et al., 2009). Therefore, measuring CNS fatigue would also improve the assessment of muscle function recovery. Non-invasive CNS assessments could use an interpolated twitch during a maximal voluntary contraction (Allen et al., 1995) but would require the use of stimulation units. Collectively, more research of this nature will help to improve understanding of how CWI influences muscle function.

Loss of muscle strength and/or power can also be caused by fatiguing contractions (i.e., short-term strength and/or power loss caused primarily by energy depletion, and/or short-term “reversible” decrements in excitation contraction coupling) or blunt force trauma—the latter of which causes damage to structural and force-generating proteins of the muscle. Therefore, restoration of muscle strength and/or power by CWI would ideally need to affect several components of excitation contraction-coupling, synthesis of essential proteins, and restoration of the muscle architecture. CWI is thought to expedite recovery from exercise by lowering skin, intramuscular and body temperature, cardiovascular strain, blood flow and increasing metabolism, blood pressure and heart rate (Bleakley and Davison, 2010b; Ihsan et al., 2016). Although CWI does not influence glycogen resynthesis rates after exhaustive exercise in humans (Gregson et al., 2013), other cryotherapy applications can reduce inflammatory cell infiltration after soft tissue injuries in animal studies (Bleakley and Davison, 2010a) and CWI can lower inflammatory biomarkers after contact sport (Lindsay et al., 2017) and resistance exercise in humans (Missau et al., 2018). However, there are equivocal findings that CWI does not affect muscle-specific or circulating inflammatory biomarkers after resistance exercise (Peake et al., 2017a), repeated sprints (White et al., 2014) or volleyball training (De Freitas et al., 2019) in humans. This variation may be attributed to the level of muscle damage imposed by the initial exercise. The first wave of responders to sites of muscle damage (strength and/or power loss) includes granulocytes, and mononucleated cells such as macrophages, eosinophils and monocytes. Considering that cold-stress limits mononuclear cell activity (Lindsay et al., 2016; Reynés et al., 2019), and inflammation is integral to muscle repair and regeneration (Peake et al., 2017b), it follows that CWI may in fact delay the sequence of events involved in muscle repair (Tidball, 2011) and the recovery of muscle strength and/or power. Additionally, CWI may slow recovery from structural protein damage, because protein synthesis, ribosomal biogenesis and anabolic signaling are temperature-dependent (Roberts et al., 2015; Figueiredo et al., 2016; Fuchs et al., 2020). Overall, the mechanisms by which CWI may affect recovery of muscle strength and/or power have not definitively been determined.

Ensuring muscle strength and power measurements are considered as a primary outcome measure for CWI studies investigating forms of exercise in which recovery of strength/power is important (independent of inflammatory status or the ultrastructural integrity of the muscle) is critical. This is because even muscle that is severely structurally compromised, with a steady state of inflammation and heightened sensitivity to exercise-induced loss of sarcolemmal excitability, can produce strength and power. For example, skeletal muscle from dystrophin-deficient mice, a model of Duchenne muscular dystrophy, undergoes continuous cycles of degeneration and regeneration, inflammation, exercise-induced loss of sarcolemmal excitability and replacement of muscle with adipose and fibrotic tissue (Tanabe et al., 1986; Baumann et al., 2020). Functional analyses indicate that absolute strength and rate of force development during a twitch and tetanic contraction of these dystrophin-deficient muscles in mice is not different to healthy skeletal muscles (Lindsay et al., 2019). However, although inflammatory status and skeletal muscle integrity might not affect muscle strength and/or power in a diseased state, it may predispose muscle of healthy individuals to greater levels of exercise-induced stress that could, in turn, lead to poorer long-term performance or extended recovery periods.

## Brief Systematic Review—CWI and Muscle Strength

Despite the variation in outcome variables and advancements in muscle strength and power assessment technologies for CWI research in humans, relatively few studies have included the measurement of muscle strength and power as a measure of the effectiveness of CWI. A literature search in PubMed identified a total of 427 peer-reviewed studies on “cold water immersion” AND “muscle” (01/12/2020). Of these 427 studies, 31 (7%) measured muscle strength prior to and following exercise and CWI. Of the 31 studies that measured strength prior to and following an intervention, 14 studies showed positive effects for CWI over a passive or active recovery modality on strength and/or power variables, six studies showed that CWI was detrimental to muscle strength and/or power, and 11 studies showed no effect. Twenty-one of the 31 studies completed only a single application of CWI, whereas 10 studies completed two or more applications of CWI. Overall, our literature search of CWI and muscle strength measurements provides conflicting evidence that CWI has beneficial effects for muscle strength variables.

## Conclusion

The efficacy of CWI has been tested and studied for decades, with large variation in outcomes. Although outcome measures remain relatively constant, the difficulty in assessing CWI as a strategy for post-exercise recovery is associated with the variability in the intervention itself. While investigating CWI protocol variables does provide additional information, it somewhat contributes to the level of confusion accompanying this modality for amateur and professional athletes. Therefore, we re-iterate that independent of the CWI protocol used in a study setting, that measures of absolute or relative muscle strength and/or power should be the primary measurement. This approach will at least offer scientists, athletes and coaches a comparison among CWI studies in the outcome variable that is relatively easy to interpret, and matters most to athletic performance.

## Author Contributions

AL conceived and wrote the opinion. JP conceived and critically reviewed the opinion. All authors contributed to the article and approved the submitted version.

## Conflict of Interest

The authors declare that the research was conducted in the absence of any commercial or financial relationships that could be construed as a potential conflict of interest.
